# 3D Muscle Architecture of the Pectoral Muscles of European Starling (*Sturnus vulgaris*)

**DOI:** 10.1093/iob/oby010

**Published:** 2019-02-01

**Authors:** S P Sullivan, F R McGechie, K M Middleton, C M Holliday

**Affiliations:** Department of Pathology and Anatomical Sciences, University of Missouri, Columbia, MO, USA

## Abstract

Avian flight is achieved through a number of modifications to the body, including the pectoral girdle, yet little is known about the architecture of the pectoral musculature. Muscle architecture is a critical variable in determining the biomechanical function of the vertebrate musculoskeletal system; however, accurate three-dimensional (3D) understanding of muscle architecture has been historically difficult to acquire. Here, we present a musculoskeletal model of a European starling (*Sturnus vulgaris*) pectoral girdle generated from iodine contrast-enhanced micro-computed-tomography (CT) data and 3D fiber tracking analysis. We used a template-based fiber-tracking algorithm to reconstruct muscle fibers in 3D based on grayscale differences in CT images, which allowed us to estimate fascicle lengths, pennation angles, muscle volumes, and physiological cross-sectional area. Our modeled muscles were qualitatively accurate; however, quantitative muscle architecture data differed between digital and traditional gross-dissection methods reflecting the complex organization of the tissue and differing natures of data collection. We found that model quality is affected by the resolution of CT image data and the fiber-tracking program’s input parameters. Nonetheless, digital fiber tracking offers numerous advantages over gross-dissection methods, most importantly, the ability to visualize and quantify entire muscles in three-dimensions, yielding a much more accurate estimation of whole muscle architecture.

## Introduction

Bird flight is an intricate symphony of muscle, bone, and connective tissue interaction, mediated by complex neuromuscular control and sensory feedback (Hedrick and Biewener 2007; Ros and Biewener 2016). Understanding flight is among the great challenges in functional morphology, given the diversity of morphology and flight style found among the 8500 extant species of flying birds. New tools and methodologies promise to increase our understanding of the association between morphology and flight behavior in any bird. Here we report on one such tool—algorithmic muscle architecture reconstruction using contrast-enhanced computed-tomography (CT)—and demonstrate its utility for rapidly acquiring anatomical data relevant to functional and evolutionary studies.

### Muscle architecture in functional morphology

Skeletal muscle is a five-level tissue: myofilament, sarcomere, muscle fiber, muscle fascicle, and whole-muscle. Muscle architecture involves the intermediate structural levels between whole-muscle morphology and sarcomere arrangement (for a review, see Lieber and Fridén 2000). In effect, it relates the shortening of individual myofilaments to contractile properties of entire muscle via the spatial arrangement of muscle fibers and muscle fascicles. The individual properties of muscle fibers (Hill 1938; Gordon et al. 1966) are aggregated by their spatial arrangement within muscle, such that a whole muscle can exhibit a range of contractile properties. Consequently, whole-muscle properties cannot necessarily be inferred from those of an isolated fascicle or, by extension, an isolated fiber or sarcomere (e.g., Moo and Herzog 2018).

Muscle architecture in relation to function was first quantified in the 20th century. Physiological cross-sectional area (PCSA)—calculated from fiber length, fiber pennation angle, and whole-muscle volume—was found to correlate with maximum isometric force production (Schumacher 1961; Gans 1982), providing a standardized way to compare maximum muscle forces within and among organisms. Tendon material properties and morphology were also found to affect contractile performance of whole muscle (Biewener and Roberts 2000). The various qualitative classifications of muscle architecture noted by early anatomists (parallel-fibered, bipennate, radial, etc.) were understood to have quantifiable functional significance in terms of contractile displacement, force, and velocity. In combination with knowledge of a muscle’s origin, insertion, and force-length behavior (which constrain contractile behavior *in vivo*), the biologically relevant performance of a muscle of given volume and architecture could be predicted. Furthermore, muscle architecture became a viable subject of evolutionary study within the scope of musculoskeletal adaptation (e.g., Gans and Gaunt 1991).

In practice, collecting muscle architecture data can be time consuming and tedious, hampering the refinement and testing of models of muscle architecture–function correspondence. Gross dissection is the primary method at the disposal of investigators. As such, it has become the *de facto* standard method of muscle architecture studies (e.g., Herrel et al., 2000; Taylor et al., 2018), although other methods are available including sonomicrometry (Brainerd and Azizi 2005) and magnetic resonance imaging (MRI) (Heemskerk et al. 2005). During dissection, a muscle is typically extracted and weighed, and its fibers are isolated to record fiber lengths. If fibers are not in parallel with the muscle’s line of action (i.e., if the muscle is pennate), pennation angles are measured with a goniometer prior to or during the muscle’s disassembly (Smith et al. 2006) or are calculated from linear measurements of the muscle belly and tendon (Anapol and Barry 1996). However, neither three-dimensional (3D) fascicle angle nor curvature can be recorded using this method. Other practical concerns include the destructive nature of dissection and difficulty obtaining comparable data from muscles of various sizes and architectures. Its inherent irreversibility makes the technique hard to justify on rare specimens, and even when specimens are abundant, its relative laboriousness limits the sample size or taxon count. Effects of scale also complicate data collection (e.g., dissections necessitating high magnification). Data such as PCSA (and its component parameters) are often not directly comparable across studies due to differing methodological details, especially sampling regimes. For example, the relative amount of fibers sampled from a muscle may vary between studies; some workers measure all fibers of a muscle (Kupczik et al. 2015), while others, the sample fibers (or fascicles as proxies) by position along an anatomical axis (Anapol and Barry 1996), by muscle compartment, or randomly across the muscle belly (Smith et al. 2006). Furthermore, some studies do not record pennation angle, especially when they are presumed to be small (Yang et al. 2015). Nevertheless, gross dissection has the advantage of being a relatively simple method for many taxa, often requiring nothing more than standard dissection tools and measurement devices. Regardless, new methodological improvements offer enormous potential for increased understanding of muscle architecture, functional morphology, and evolutionary biology.

### Digital muscle architecture reconstruction

Digital visualization methods are now able to provide easier access to quantitative muscle architecture data, while also vastly increasing the types and amount of data that can be gathered. These methods range from hybrid approaches, using a combination of dissection and digital data collection (e.g., serial cryosectioning; Stark and Schilling 2010; Otake et al. 2018), to fully digital, non-invasive approaches (Orsbon and Ross 2018). Ultrasonography allows for architectural parameters such as pennation angle and two-dimensional (2D) fiber length to be collected in real-time on living (Rutherford and Jones 1992; Maganaris and Baltzopoulos 1999) or deceased subjects (Narici 1999). However, data collection is generally restricted to a small and superficial viewing area of relatively large specimens and regional variation in 3D morphology cannot generally be obtained (Heemskerk et al. 2005; but see Rana et al. 2018), making it non-ideal for many functional and comparative studies. Digital reconstruction of whole muscle in 3D can be collected using digitizing microscribes, MRI, and contrast-enhanced CT. Microscribe methods involve digitizing the paths of individual fascicles *in situ* during the layer-wise, manual dissection of a muscle (Lee et al. 2015; Nyakatura and Stark 2015; Rosin and Nyakatura 2017). This results in a schematic but high-fidelity 3D muscle model, although the effort and time required is substantial, especially for larger muscles (Rosin and Nyakatura 2017). Finally, MRI coupled with manual segmentation (Infantolino et al. 2012) or diffusion tensor imaging (Damon et al. 2002; Heemskerk et al. 2009; Heemskerk et al. 2010; Damon et al. 2017; Lansdown et al. 2007) has been successfully employed to visualize and reconstruct 3D muscle architecture using computational approaches.

CT-based imaging is now widespread in the study of vertebrate anatomy, and the latest technique to benefit from automatic fiber-tracking methods. The ability to image soft-tissues using contrast-enhanced CT methods greatly increased the utility of CT imaging, adding to its cost and resolution advantages over MRI, especially on small organisms (e.g., diffusible iodine contrast enhanced microCT [DiceCT]: Metscher 2009; Gignac et al. 2016). As a result, 3D muscle anatomy has now been reported for jaw muscles (Cox and Jeffery 2011; Jeffery et al. 2011; Baverstock et al. 2013; Holliday et al. 2013; Lautenschlager et al. 2014) and limb muscles (Bribiesca-Contreras and Sellers 2017), among other muscles. Like non-diffusion-tensor MRI, these studies involve manual segmentation to produce volumetric models. Recently, because iodine staining produces contrast between individual myofibers (Jeffery et al. 2011), workers have applied texture-based fiber-tracking algorithms to model and visualize cardiac muscle (Aslanidi et al. 2012) and skeletal muscle (Kupczik et al. 2015; Dickinson et al. 2018; for a fiber-tracking method using non-contrast-enhanced CT, see Otake et al. 2017). A related fiber reconstruction method, template-based fiber-tracking, has been used on biological data (cellular structure: Rigort et al. 2012; Weber et al. 2012; intervertebral disc morphology: Disney et al. 2017), but until now has not been tested on skeletal muscle.

### Present study

Heeding the call for more and novel uses of visualization in the sciences broadly (Munzner et al. 2006) and within avian morphology specifically (James 2017), we present digital models of the pectoral musculature of a European starling (*Sturnus vulgaris*) using a template-based fiber-tracking algorithm. In evaluating the method, we also present a systematic exploration of the parameter space of template-based muscle architecture reconstruction. We chose to demonstrate our new method on the avian pectoral system because of its functional relevance to flight and the substantial architectural variation in pectoral muscle morphology.

The aim of this paper is two-fold: (1) to present a 3D model of pectoral and brachial muscles and bones, including visible *in situ* muscle fascicle architecture and (2) to explore the use of template-based fiber-tracking programs in reconstructing skeletal muscle architecture generally. To the latter end, we performed a sensitivity analysis of the fiber tracking program Avizo Xfiber (FEI Visualizations Science Group; Hillsboro, Oregon) and compared the quantitative results to those gathered from a traditional dissection-based approach.

## Materials and methods

### Terminology

Terminology of myological organization varies among studies and applications and has resulted in some confusion as to what we are measuring when studying “muscle architecture.” The word “fiber” is used in numerous ways, both as a general term to describe strands of material (fiber optics, collagen fibers, threads in textiles, etc.) and as a specific anatomical term. Technically, muscle fibers, or myofibers, are individual muscle cells which are encapsulated by endomysium and are generally only 1–4 µm in diameter (e.g., Slavin et al. 1982). Muscle fascicles are bundles of muscle fibers packaged in perimysium and are generally an order of magnitude larger in diameter than muscle fibers. We think that we are seeing and recording fascicles in 50 µm voxel size-contrast images of vertebrate skeletal muscles. We also think that our 3D reconstructions are those of muscle fascicles, despite using ‘fiber’ tracking software (e.g., Xfiber), or conducting a study of muscle ‘fiber’ architecture as well as reporting on 3D muscle ‘fibers’. This article seeks to find congruence between organic, *ex vivo* muscle fascicles and *in silico*, modeled muscle ‘tracts’.

### Specimen information

**Fig. 1:**
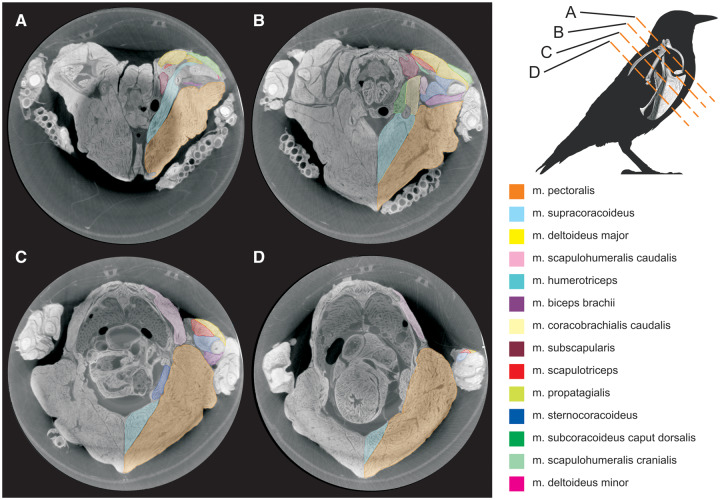
Transverse DICE-CT slices of starling (*S. vulgaris*) pectoral region. Slice locations are indicated in the top-right diagram.

A mature, wild-caught European starling (henceforth, starling) was used in this study. The specimen had initially been collected in 2001 for use in an EMG study on hindlimb digital muscles (Middleton 2003), after which it was euthanized and frozen; it remained frozen for ∼15 years before its use here. Apart from some compression of the superficial pectoral muscles by the folded wings (Fig. 1), the specimen was intact. We deemed the use of this “non-ideal” specimen acceptable because its gross morphology was indistinguishable from recently-dead starling specimens, and because our analyses and method comparison only involved the single specimen in order to assess method correspondence irrespective of specimen quality. Furthermore, we anticipate future workers will apply our digital method to similarly-stored specimens. Based on our results, Xfiber can still produce plausible muscle fascicle models from long-frozen specimens.

Prior to further processing, the specimen was skinned and its distal hind limbs, head, and cranial-most cervical vertebrae were removed. Care was taken not to disturb the pectoral region. The body was fixed in 10% neutral-buffered formalin for 2 weeks, then stored in 70% ethanol prior to scanning and staining.

### Scanning and staining

The specimen was scanned twice under different preparations to facilitate the visualization and segmentation of mineralized (bone) and non-mineralized (muscle, tendon) tissues. We first scanned the unstained specimen to reconstruct skeletal morphology (80 kV, 500 mA, 83 µm voxel size; Siemens Inveon MicroCT, Munich, Germany). The specimen was subsequently immersed in a 10% iodine potassium iodide (I_2_KI; Lugol’s iodine; Carolina Biological Supply Company, Burlington, North Carolina) solution for 34 days to enhance soft tissue contrast (Metscher 2009; Holliday et al. 2013; Gignac et al. 2016). Following contrast, the specimen was scanned with an Xradia 510 Versa microCT scanner (Carl Zeiss AG, Oberkochen, Germany; 140 kV, 10 W, 43.863 µm voxel size).

### Segmentation and volume model

All microCT images were imported as .tiff files into Avizo 9.4 Lite (FEI, Thermo Fisher Scientific) for segmentation. On the unstained specimen images, we primarily used the Magic Wand thresholding tool with manual adjustment (using the Brush tool) as needed. We extracted the entire skeletal pectoral girdle as 3D surfaces within Avizo, then simplified each element mesh in GeoMagic Studio 13 (Geomagic, Inc., Research Triangle Park, NC). Due to the decreased contrast between hard and soft tissues (primarily bone and muscle, respectively) in the stained specimen images, soft tissues were manually segmented using the Brush tool, interpolating between 3 and 10 slices. Although bones were difficult to resolve in the contrast-enhanced specimen, we reconstructed several to aid in muscle identification and segmentation. Tendon, and the connective tissue layers that surround muscle tissue, in general, was hard to distinguish from muscle fascicles and fibers; notable exceptions were the relatively large central tendons of m. supracoracoideus and m. pectoralis.

To make a clean hard- and soft-tissue volume model, we combined the bone volumes from the unstained scan with the soft-tissue volumes from the stained scans in Avizo. Transformation of volumes from both datasets was needed to register the models because of postural shift between scans and dissimilar image axes.

### Muscle architecture modeling

**Fig. 2:**
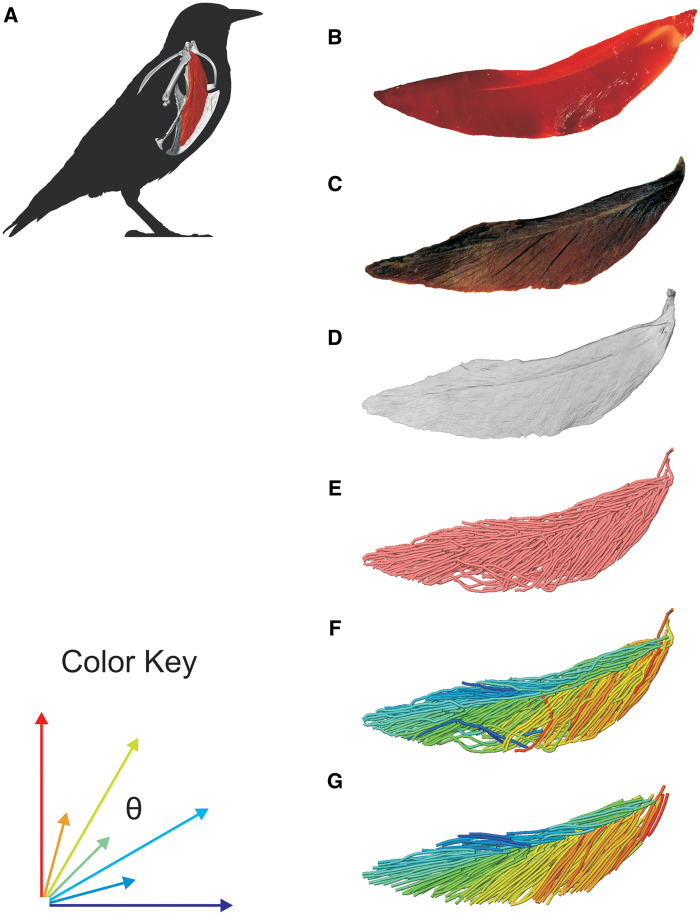
Diagrammatic workflow of volume and tract-model creation. All muscle photographs and illustrations depict a lateral view of the right m. supracoracoideus. (**A**) Starling (S. vulgaris) silhouette indicating the location of the right m. supracoracoideus within the pectoral girdle. (**B**) Fresh muscle. (**C**) Muscle after formalin fixation and contrast-enhancement via iodine potassium iodide. (**D**) Segmented muscle volume rendering. (**E**) Initial Xfiber fascicle-tract model. (**F**) Model in (**E**) colored by tract orientation. (**G**) Final, edited model. Colors in (F) and (G) represent angle θ, a proxy for pennation angle, which ranges from 0° to 90° (see Color Key).

We used the Avizo 9 extension, Xfiber, to reconstruct shoulder muscle architecture from the contrast-enhanced CT data (Fig. 2). Xfiber uses a template-based, probabilistic, automatic fiber-tracking program to reconstruct fascicle paths from segmented whole-muscle volumes. The program is especially sensitive to underlying image quality (Westenberger and Blanc 2016), because it uses grayscale variation between individual voxels to estimate the trajectory of putative fibrous structures, whose centerlines it traces. For our purposes, this means that the program requires consistent contrast differences between intramuscular connective tissues and muscle fascicles and a sufficiently small voxel size such that these tissues can be resolved (see section “Discussion”). The latter requirement was met by the <50 µm voxel size, while the former was met by using iodine as a contrast agent, which selectively binds to and increases contrast in many soft tissue structures. Iodine binds to glycogen, which is contained in myofibers but not the sheathing connective tissues endomysium and epimysium, thereby revealing boundaries between adjacent myofibers and fascicles (Jeffery et al. 2011).

The basic steps followed within Xfiber are (1) the manual setting of a cylindrical template, (2) the propagation of that template cylinder through the image data to produce a probabilistic 3D map of the likelihood of fit between the cylinder template and the data, and (3) the tracing of visible tracts on that map according to various user-set parameters. Furthermore, once tracts are traced, individual tracts can be edited (e.g., deleted) manually.

Segmented muscles, saved as NIfTi (.nii) objects, were isolated as individual image stacks with all extramuscular material set to a grayscale value of zero. We applied Xfiber's Cylinder Correlation module to each muscle (step 1), which sets a cylindrical template according to various parameters relating to the dimensions of the template cylinder and the subsequent propagation of that cylinder through the image data. Of note is the “Outer Cylinder Radius” parameter, which was set to the diameter of a typical fascicle (65–140 µm) as measured in the image data. Two output objects result from the Cylinder Correlation module (step 2)—an orientation field (essentially a vector cloud describing fiber orientation throughout the muscle volume) and a correlation field (a probability field describing the fit of the cylinder template to the image data).

To remove boundary artifacts of the muscle objects caused by human error in manual segmentation, we first cropped muscle label fields using the Avizo Erosion module, and then combined the cropped label field with the correlation field using the Arithmetic module. This step also prevents Avizo from reconstructing spurious tracts outside of the original muscle volume in the tracing step. These tracts may form when sharp grayscale transitions exist in an image stack, such as between the segmented muscle and surrounding black volume.

We combined the cropped correlation field with the orientation field with the Xfiber Trace Correlation Lines module, which traces tracts according to the matrix formed by the intersection of the two fields. User-defined parameters affect how tracts are traced within the matrix, including minimum length, maximum curvature, and other tracing criteria. Traced tracts were output as correlation lines objects and visualized using the Spatial Graph View module, which allows fiber objects to be scaled and colored according to various parameters. We tailored Xfiber parameters for each muscle using values that produced qualitatively plausible models (Fig. 4; Supplementary Table S1). Highly inaccurate tracts (e.g., travelling perpendicular to the known fascicle direction or containing >90° bends) were individually deleted after comparison to the DiceCT data, and the remaining tracts were smoothed.

### Xfiber sensitivity analysis

We ran a sensitivity analysis of Xfiber to explore the application of the program to muscle tissue—not only to forming qualitatively accurate fascicle models, but also to extracting quantitative muscle architecture data comparable to traditional, dissection-based methods. We limited our analysis to a single muscle, the right m. supracoracoideus. This muscle was selected because it remained naturally intact in our specimen (e.g., compared to the superficially compressed m. pectoralis), and it has complex but well-documented muscle architecture in starlings and many other taxa. Its intermediate size, between m. pectoralis and all other pectoral muscles, also aided in its virtual and actual dissection.

Generally, we tested the effects of different parameter values on quantitative and qualitative muscle architecture reconstructions. Twelve default parameters of the Cylinder Correlation, Erosion, and Trace Correlation Lines modules were adjusted until the m. supracoracoideus fascicle model reasonably resembled a starling m. supracoracoideus as known from previous starling dissections (i.e., in lateral view, roughly fusiform with pennate fibers passing posterodorsally and posteroventrally from a central tendon). This “target” trial was set as a baseline for comparisons (referred to as the “baseline run”). These 12 parameters were then altered (increased and decreased) relative to the baseline parameters to produce 48 more models, exploring the parameter space of fascicle architecture estimation (see Fig. 4). The increments between parameter values for each of the trials were selected such that the most extreme values were themselves unrealistic or produced obviously unrealistic fascicle models, thus exploring the complete range of reasonable values for each parameter.

Variables relevant to the calculation of PCSA—volume, fascicle length, and pennation angle—were extracted from each of the trials for comparison to dissection-derived estimates. Volume was constant for all trials. Individual fiber tract data and statistics for fascicle length and pennation angle were exported from the correlation lines object of each trial. Xfiber reports both the curved lengths and chord lengths of reconstructed tracts—we used curved lengths. The orientations of individual tracts, reported in spherical coordinates within Avizo, were used to estimate pennation angle. We transformed the original muscle image data such that the average long-axis orientation of the internal m. supracoracoideus tendon was aligned with the z-axis (keeping the caudal m. supracoracoideus more positive to measure acute angles). The angle theta (θ), defined as the angle from the positive *z*-axis, thus estimated the acute angle between the internal m. supracoracoideus tendon and its attached fibers.

### Traditional muscle architecture analysis

To prepare the specimen for dissection, we leached most of the visible I_2_KI stain from the specimen by immersing it in 70% EtOH for ∼5 months. The ethanol was changed as needed, until the specimen largely returned to its original color. The specimen appeared grossly unaltered from its original state, although muscles were nominally less pliable and bones were significantly less rigid than prior to fixation. The effects of iodine staining on fiber/fascicle length and pennation angle are unknown, although shrinkage and deformation in various tissues have been documented (Vickerton et al. 2013; Balint et al. 2016; Disney et al. 2017; Santana 2018; but see Baverstock et al. 2013). Via dissection, we obtained muscle volume, pennation angle, and fascicle length data for the right m. supracoracoideus to compare with the digital models.

We removed the right m. supracoracoideus entirely, cutting the tendon at its humeral insertion. After blotting the muscle dry with a paper towel, the muscle was weighed to the nearest 0.0001 g. We converted the mass to volume using a muscle density of 1.055 g/cm^3^ (obtained from fixed human muscle; Ward and Lieber 2005). Rather than gathering data on all visible fascicles of the m. supracoracoideus, we chose a more typical sampling regime. To measure pennation angle, 30 fascicles, evenly spaced along the length of the muscle belly, were photographed *in situ* under magnification. We measured pennation angle in ImageJ (https://imagej.nih.gov/ij/), defined here as the angle between each fascicle proximal to the tendon and the central tendon itself, in the plane of the image, using light dissection to reveal tendon location when necessary. To isolate individual fascicles for length measurements, we digested the muscle’s connective tissues in a 30% nitric acid solution for 5 h. After careful dissection spanning the entire belly, 50 fascicles were isolated from the muscle belly and photographed over grid paper. We used the curve measuring tool in ImageJ to measure 2D fascicle length. Tendinous fascicle ends were not included in the length measurements.

### PCSA calculation

We calculated PCSA from our dissection- and Xfiber-derived muscle architecture data using the formula: (muscle volume (cm^3^) * cos(θ))/fascicle length (cm)), where θ is pennation angle. We did not scale fascicle lengths using a sarcomere correction factor (Anapol and Barry 1996).

## Results

### Starling shoulder volume and muscle architecture reconstructions

**Fig. 3:**
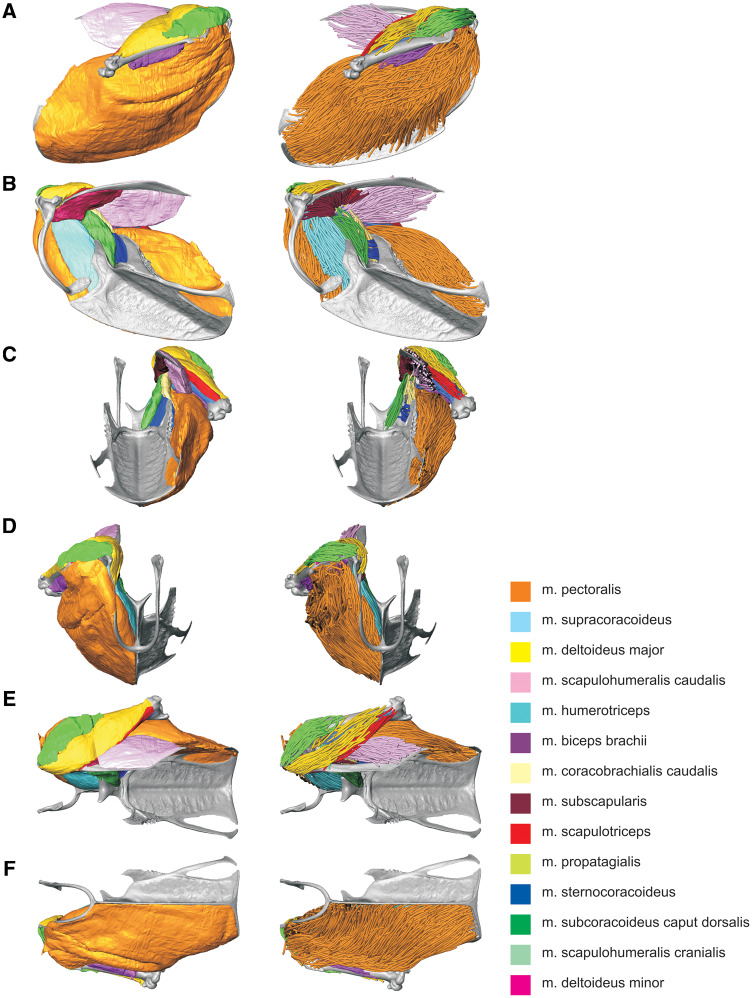
Starling (S. vulgaris) shoulder and pectoral musculoskeletal models. Muscles are represented as volumes (left column) and fascicle tracts (right column). (**A**) Lateral view. (**B**) Medial view. (**C**) Caudal view. (**D**) Rostral view. (**E**) Dorsal view. (**F**) Ventral view. Muscle colors as in Fig. 1.

We reconstructed an entire right shoulder of the starling from our stained and unstained CT data (Fig. 3). Fourteen muscles (Table 1) and four partial bones were segmented and isolated from the stained specimen images, while the complete sternum, furcula, and right humerus, coracoid, and scapula were segmented from the unstained specimen images. All structures described in a previous study of the starling shoulder (Dial et al. 1991) were resolved except for the os humerocapsularis, although we confirmed its presence in the specimen during dissection. Apart from the unnaturally undulating m. pectoralis surface—an artifact of tying the wings to the thorax for immersion and scanning—all muscles appeared normal and consistent with existing descriptions of starling or passerine anatomy. The volume model was also checked against the actual specimen, revealing no notable differences in gross shape, muscle attachments, or relative volumes. Note that the distal-most regions of m. biceps brachii and m. humerotriceps were not sufficiently resolved in the CT data and were therefore not segmented. Volume estimates of these two muscles are likely marginally smaller than their true volumes.

**Table 1:** Segmented muscle volumes

Muscle	Volume (cm^3^)
Pectoralis	5.178
Supracoracoideus	0.690
Deltoideus major	0.323
Scapulohumeralis caudalis	0.313
Humerotriceps	0.172
Biceps brachii	0.121
Coracobrachialis caudalis	0.107
Subscapularis	0.105
Scapulotriceps	0.097
Propatagialis	0.091
Sternocoracoideus	0.074
Subcoracoideus caput dorsalis	0.073
Scapulohumeralis cranialis	0.011
Deltoideus minor	0.002

**Fig. 4:**
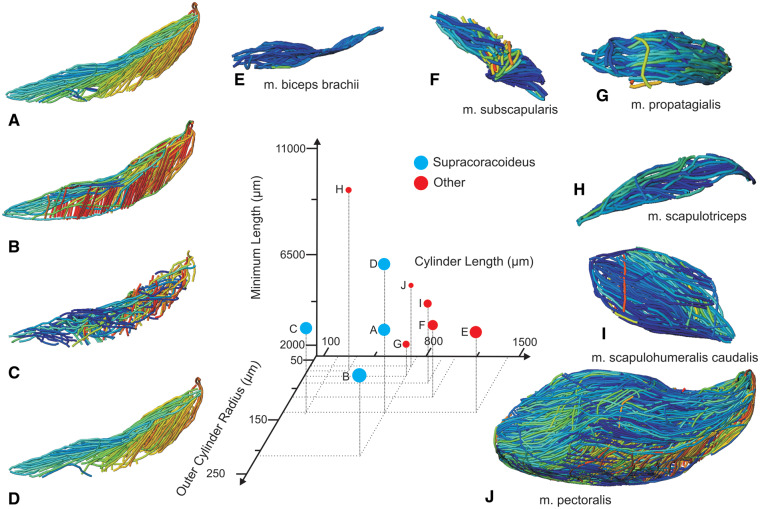
Unedited muscle architecture models plotted within a 3D parameterspace: (Cylinder Length, Outer Cylinder Radius, Minimum Length). (**A**) m. supracoracoideus (700, 140, 5600), (**B**) m. supracoracoideus (700, 210, 5600), (**C**) m. supracoracoideus (140, 140, 5600), (**D**) m. supracoracoideus (700, 140, 8400) (**E**) m. pectoralis (1400, 140, 5600), (**F**) m. scapulohumeralis caudalis (1000, 110, 5000), (**G**) m. subscapularis (700, 75, 3000), (**H**) m. biceps brachii (350, 70, 10000), (**I**) m. scapulotriceps (900, 90, 5600), (**J**) m. propatagialis (700, 65, 5600). Muscles are in lateral view. Models not to scale. Model tract colors as in Fig. 2.

The complete fiber-tracked shoulder (Figs. 3, 7–22) also appeared grossly consistent with the qualitative muscle architecture descriptions in Dial et al. (1991), although certain muscles did contain more obvious artifacts than others prior to manual cleaning. Individual tracts were not always consistent with fascicle paths as visible within the DiceCT images or as observed via gross dissection. Rather, reconstructed tracts were often observed to follow several adjacent fascicle paths. The resultant models thus contained some fascicles that crossed paths and strayed from dissection-observed trajectories prior to manual cleaning. Because we only collected quantitative muscle architecture data for m. supracoracoideus; the usefulness of the other muscle fascicle models for functional modeling is unknown. Nevertheless, the general architecture of every muscle could be quickly and easily recognized from the cleaned models (Fig. 3).

### Xfiber sensitivity analysis

**Fig. 5:**
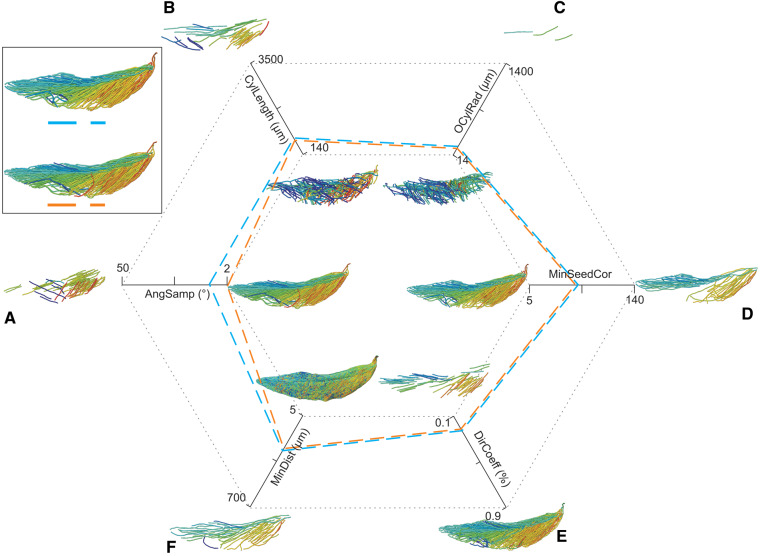
Illustration of the sensitivity analysis. Parameters were individually increased and decreased relative to the baseline target model. Smaller models represent the extremes of each parameter’s indicated range with other parameters set constant at the values indicated by the blue dashed line. Boxed models depict the optimized model (orange dashed line) and the baseline target model (blue dashed line) prior to manual tract editing and smoothing. (**A**) Angular Sampling (AngSamp), (**B**) Cylinder Length (CylLength), (**C**) Outer Cylinder Radius (OCylRad), (**D**) Minimum Seed Correlation (MinSeedCor), (**E**) Direction Coefficient (DirCoeff), (**F**) Minimum Distance (MinDist).

All 49 Xfiber fascicle models made for the sensitivity analysis were visually and quantitatively inspected (Fig. 5, Supplementary Figs. S1, S2). Overall, the models spanned a large range of apparent plausibility. For many parameters, intermediate values produced the most plausible models, but some (e.g., Angular Sampling) improved toward one extreme. The lowest values of Angular Sampling, as expected, produced the most plausible models, visually and quantitatively, but also required the longest processing times (10 h for data-sampling in 2° increments, compared to ∼20 min for any higher values). However, the estimates of muscle architecture (fascicle length and pennation angle) of obviously implausible models were not always the farthest from dissection data for a given range of parameter values. In other words, the relationship between visual model plausibility and quantitative similarity to dissection estimates was not predictable. Also, models with the highest fidelity to dissection estimates in one measure did not always have similar fidelity in the other measure (e.g., Trace Length). More generally, pennation angle and fascicle length did not reliably covary with increasing parameter values for most parameter trials. Despite this moderate unpredictability in the results, relatively small variation in quantitative measures accompanied many parameter changes. Few data estimates exhibited medians outside the interquartile range of the baseline run for any parameter value (Supplementary Figs. S1, S2).

### Quantitative method comparison

**Fig. 6:**
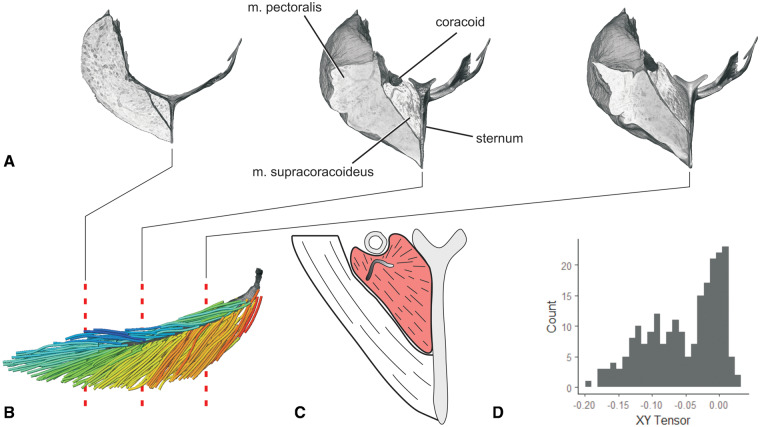
Fascicle architecture and tendon morphology of m. supracoracoideus revealed through DICE-CT and Xfiber. (**A**) Transverse slices in rostral view through the DICE-CT volume models of m. pectoralis, m. supracoracoideus, sternum, and right coracoid. (**B**) Fascicle model of m. supracoracoideus with volume reconstructed central tendon in lateral view. Dashed lines indicate slice locations in (A). Color as in Fig. 2. (**C**) Illustration of intermediate slice in (A), showing the non-planar m. supracoracoideus central tendon. (**D**) XY tensor plot of fascicle model in (B). With the Z axis aligned with the central tendon’s long axis, the XY tensor approximately reflects the radial distribution of fascicles around the central tendon.

We used muscle architecture data from the optimized m. supracoracoideus model (Figs. 2G, 6B) for comparison to dissection estimates of pennation angle, fascicle length, volume, and PCSA (Table 2). The dissection estimate of pennation angle was modestly smaller than the Xfiber estimate (mean of 18.7° vs. 23.5°), while dissection estimated longer fascicle lengths (1.27 cm vs. 1.03 cm). Volume estimations differed modestly; the Avizo volume estimate was 30% larger than from dissection (0.69 cm^3^ vs. 0.53 cm^3^), likely due to tissue shrinkage from ethanol storage prior to dissection and tissue loss during dissection. PCSA values between methods differed as well. Xfiber-estimated PCSA (0.614 cm^2^) was 48% larger than the dissection estimate (0.416 cm^2^), largely driven by the greater volume and shorter fascicle lengths estimated for the Xfiber model (see equation for PCSA). However, it remains to be tested if these are functionally significant differences in net muscle force on the humerus.

**Table 2:** m. Supracoracoideus method comparison muscle architecture data

	Avizo Xfiber	Dissection
Mass (g)	0.728	0.5895
Volume (cm^3^)	0.690	0.5588
Fiber length (cm)	1.030 ± 0.360 (N = 218)	1.271 ± 0.367 (N = 50)
Pennation angle (°)	23.53 ± 12.464 (N = 218)	18.71 ± 8.79 (N = 30)
PCSA (cm^2^)	0.61	0.42

*Note*: Values are represented as mean ± standard deviation.

**Fig. 7:**
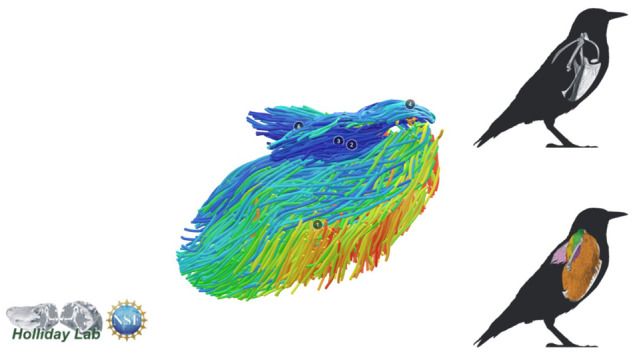
Complete pectoral and shoulder muscle fascicle model colored by fascicle orientation.

**Fig. 8:**
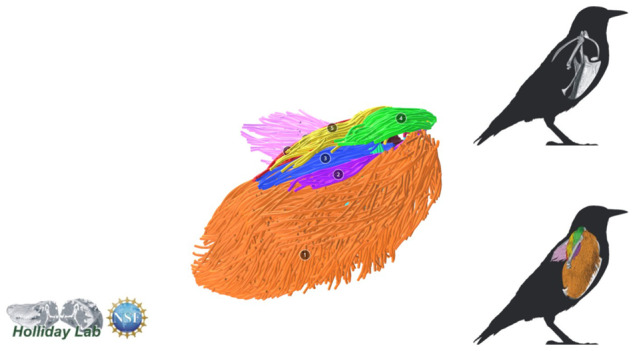
Complete pectoral and shoulder muscle fascicle model colored by muscle.

**Fig. 9:**
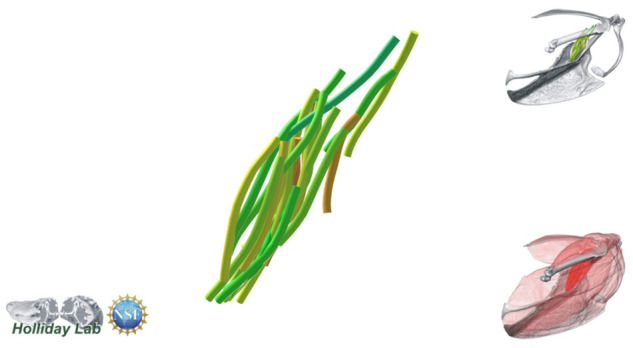
m. Coracobrachialis caudalis fascicle model.

**Fig. 10:**
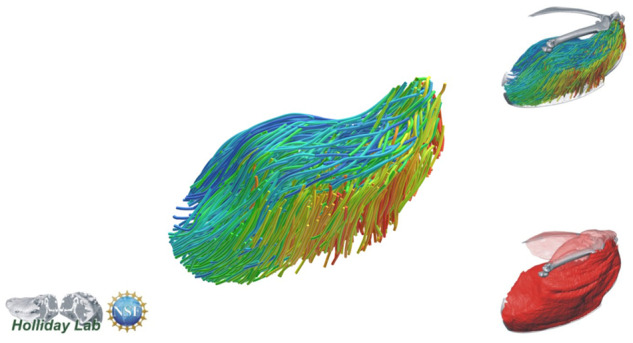
m. Pectoralis fascicle model.

**Fig. 11:**
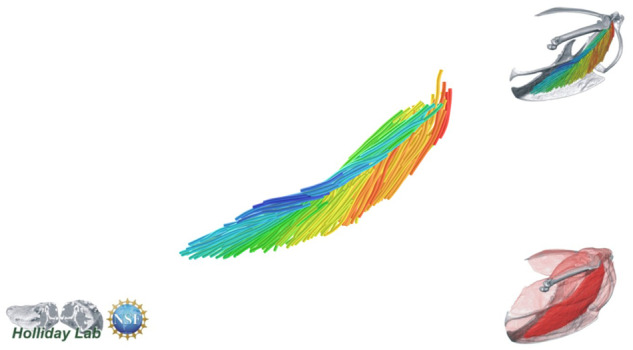
m. supracoracoideus fascicle model.

**Fig. 12:**
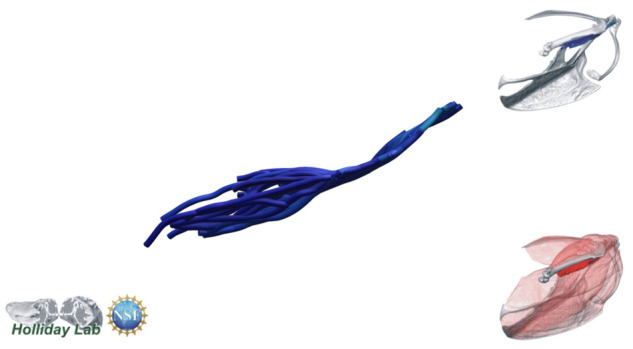
m. biceps brachii fascicle model.

**Fig. 13:**
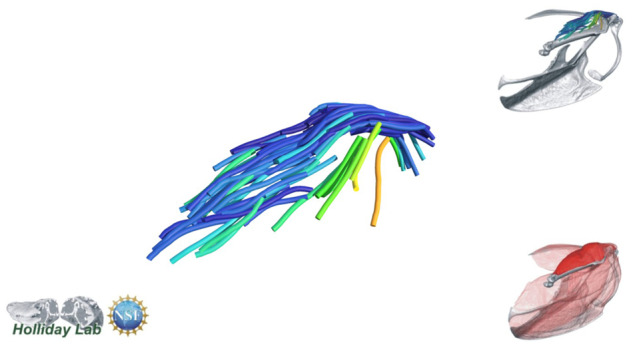
m. deltoideus major fascicle model.

**Fig. 14:**
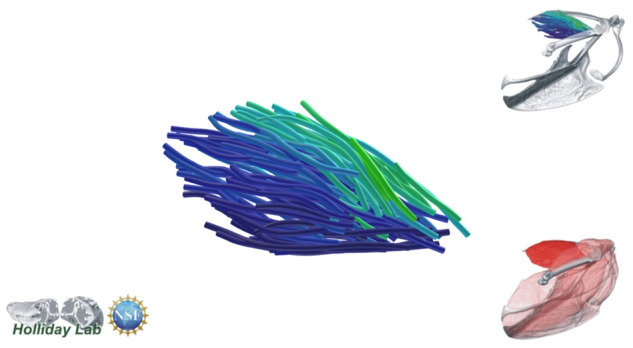
m. scapulohumeralis fascicle model.

**Fig. 15:**
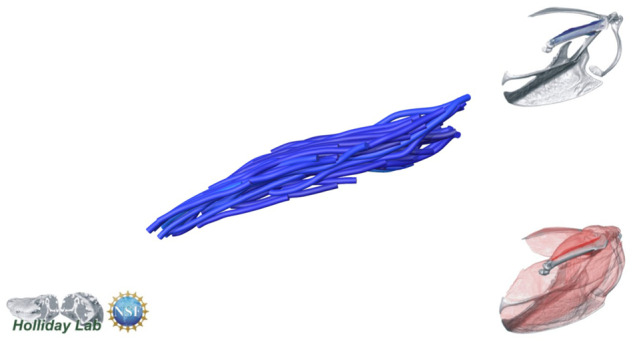
m. humerotriceps fascicle model.

**Fig. 16:**
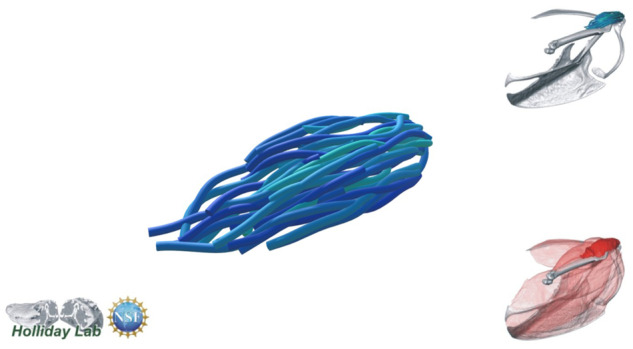
m. propatagialis fascicle model.

**Fig. 17:**
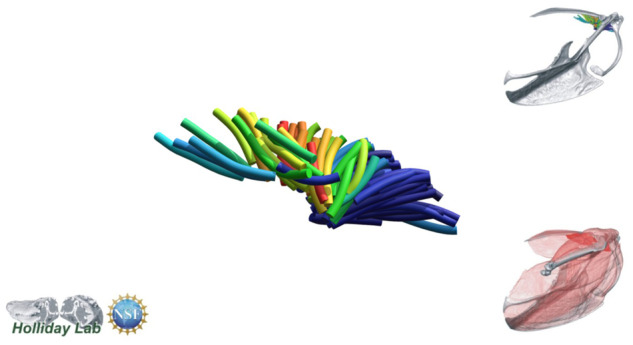
m. subscapularis fascicle model.

**Fig. 18:**
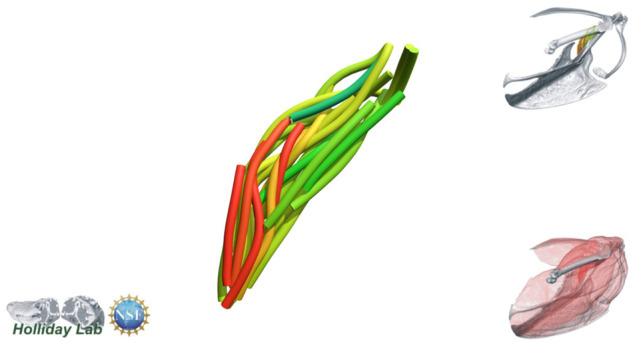
m. subcoracoideus fascicle model.

**Fig. 19:**
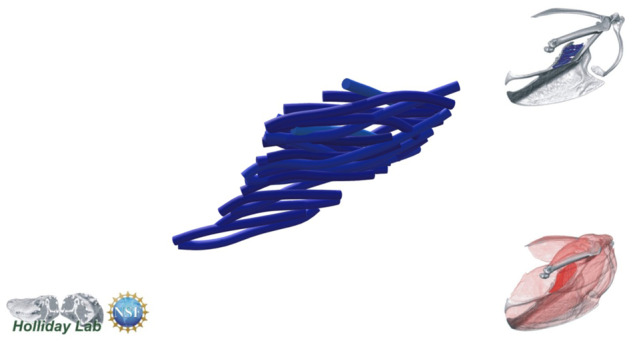
m. sternocoracoideus fascicle model.

**Fig. 20:**
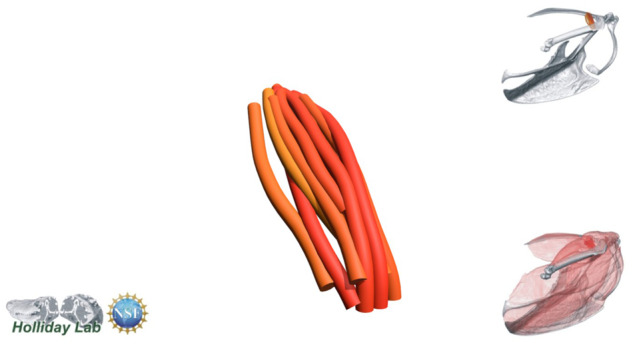
m. scapulohumeralis cranialis fascicle model.

**Fig. 21:**
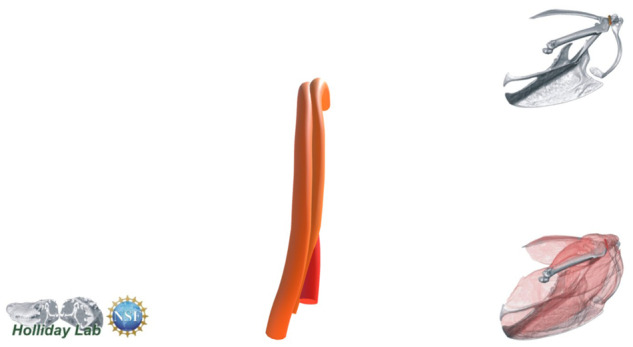
m. deltoideus minor fascicle model.

**Fig. 22:**
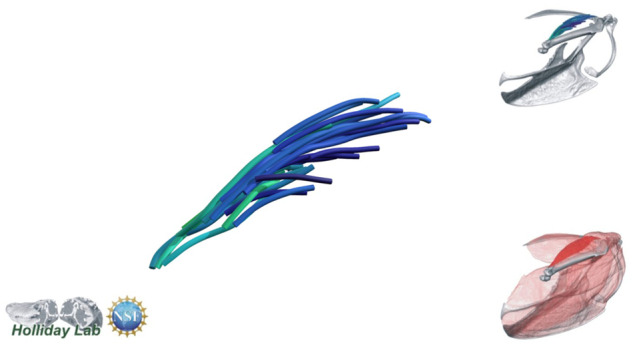
m. scapulotriceps fascicle model.

## Discussion

We digitally reconstructed starling pectoral and shoulder musculature with an emphasis on the use of Avizo Xfiber to model muscle architecture from DiceCT data. We found that Xfiber’s template-based fiber-tracking algorithm produced plausible models of muscle architecture, comparable to dissection-based methods, despite our specimen’s storage history. Interestingly, the two approaches offer different perspectives on the architecture of the tissues.

### Volume model and measurements

Our study adds to the small number of avian DiceCT studies (Düring et al. 2013; Lautenschlager et al. 2014; Li et al. 2015; Gignac et al., 2016; Bribiesca-Contreras and Sellers 2017) and is the first to involve the shoulder. The external morphologies and attachments of our segmented muscle volumes appeared consistent with direct anatomical investigation and prior studies of starling myology (Dial et al. 1991).

Some tendons were visualized despite iodine’s lower affinity for tendon than myofiber (Bribiesca-Contreras and Sellers 2017). Internal tendons were indirectly visualized as darker regions within muscle bellies (Fig. 6A); however, some large external tendons were directly visualized owing to their apparent higher stain affinity than surrounding extramuscular tissues (e.g., external portion of the m. supracoracoideus central tendon; Fig. 6B).

### Muscle architecture modeling—advantages

A 3D digital muscle architecture modeling presents an attractive and informative way to visualize muscle architecture beyond what is possible with traditional dissection and 2D illustration. Many general advantages of CT-based muscle architecture methods—namely, being non-destructive, relatively quick, repeatable, *in situ*, and fiber- or fascicle-specific—have been discussed elsewhere (Aslanidi et al. 2012; Kupczik et al. 2015; Otake et al. 2017; Dickinson et al. 2018), and also describe the Xfiber-based method demonstrated here. Xfiber, however, has additional benefits including the ability to directly check reconstructed tracts against CT images and segmented volumes; complete workflow encapsulation within Avizo, from CT image segmentation to manual refinement and visualization of volume and tract models; a transparent and highly-configurable modeling algorithm; and substantial visualization potential, especially in combination with 3D online viewing platforms such as Sketchfab (https://sketchfab.com/).

The vast visualization and illustration potential of 3D models of muscles is demonstrated by our exemplary upstroke muscle, m. supracoracoideus. m. supracoracoideus is commonly described as a classic bipennate muscle (e.g., Poore et al. 1997; Biewener 2011)—that is, with two sets of fibers inserting onto a central tendon with characteristic pennation angle. Such architecture is suggested from a ventrolateral view of the muscle, in which the central tendon is conspicuous (Fig. 2C). However, our 3D Xfiber model combined with a reconstructed tendon (Fig. 6) reveals that the internal tendon terminates medially prior to contacting the sternum. Its fascicle architecture is thus better described as hemiradial with position-dependent variation in pennation angle. Indeed, a tensor plot representing fascicle orientations about the long axis of the m. supracoracoideus belly approximates a skewed normal distribution (Fig. 6D).

### Muscle architecture modeling—limitations

We also discovered several limitations to our method, some of which apply to other digital modeling methods as well. These can be divided into those inherent to template-based fiber-tracking programs generally, and those specific to modeling quantitative muscle architecture.

#### Variable fiber diameter

A disadvantage of template-based fiber-tracking algorithms is their optimization for tracking cylindrical fibers of fixed diameter. The image texture, rather than being compared only to itself as is the case in texture-based (Kupczik et al. 2015) and tensor-voting (Loss et al. 2012) algorithms, is surveyed for grayscale heterogeneity that matches a specified template. The resulting probability field upon which fiber tracts are traced is thus a map of likelihoods that a set of voxels represents a template-like structure. Xfiber was designed originally for application to actin and microtubule networks (Rigort et al. 2012; Weber et al. 2012), whose constituent fibers have relatively consistent diameters, and was optimized for industrial fiber analytics involving homogeneous fibers (Westenberger and Blanc 2016). But skeletal muscle fibers and fascicles do not necessarily have fixed diameters within vertebrate taxa, specimens, and even individual muscles. Regarding birds, pigeon m. pectoralis myofiber diameters are known to vary intramuscularly (Young et al. 1990), and avian muscle may have a higher proportion of short, overlapping myofibers than other taxa (Gans and Gaunt 1992). As a result, even if all connective tissue is fully contrasted against myofiber or fascicle (i.e., optimal contrast enhancement), the issue of tapering and variable-diameter fibers and fascicles remains. Further, we observed apparent fascicle diameter variation in some of our specimen’s pectoral muscles through inspection of the DiceCT data. A critical question is thus what template should be used for a given distribution of myofiber or fascicle diameters in a whole muscle? Perhaps in the case of avian muscle, or skeletal muscle generally, it is best to use entirely non-template-based fiber tracking algorithms, like those used in Image3D (Kupczik et al. 2015; Dickinson et al. 2018) and other programs (Krause et al. 2010; Loss et al. 2012; Otake et al. 2017; Otake et al. 2018). Nevertheless, for objects containing complex fiber distributions (like many skeletal muscles), Blanc and Westenberger (2017) found that template-based algorithms performed better than texture-based algorithms in estimating fiber orientation. However, their abiologic dataset included only fibers of equal diameter, and it remains unclear how much diameter variation is acceptable for accurate fiber or fascicle models from template-based or non-template-based algorithms. Template-based algorithms can overcome these issues if variable-diameter template cylinders are employed, thus minimizing the inaccurate reconstruction of myofibers or fascicles whose morphologies stray from a cylindrical ideal.

#### Image resolution

Increasing resolution is not simply a matter of minimizing voxel dimensions, but also of optimizing staining protocol for even contrast between adjacent fascicles throughout their lengths and across entire muscle volumes. Adjacent fascicles that are not sufficiently contrasted, indicated by a lack of darker voxels between them, are effectively treated like fibrous structures with highly variable diameters in the Xfiber algorithm. Insufficient contrast has the tendency to exacerbate the issue of diameter heterogeneity across and within individual fibers or fascicles and may explain most instances of implausible fascicle reconstruction seen here. Most artifactual tracts in our models terminated where no contrast between adjacent fascicles existed, and dense regions of fascicles (with minimal contrast between them) often produced artificially short and/or incorrectly oriented tracts. Further, varying the template cylinder diameter (Outer Cylinder Radius) had relatively marked effects on the plausibility of models and muscle architecture variable estimates (Fig. 5C, Supplementary Figs. S1, S2).

Overall, our results suggest that the greatest accuracy may be obtained with (1) well stained and contrasted tissues and (2) relatively homogenous fascicle diameters. However, we emphasize that these algorithms need not accurately reconstruct the individual structures of interest to produce quantitatively accurate and biologically-informative results. Instead, algorithm parameters can be adjusted to produce a “good enough” abstraction of fibers or fascicles for extracting accurate muscle architecture information.

#### Quantitative muscle architecture

Both the sensitivity analysis of Xfiber models and the differences between Xfiber and dissection estimates of PCSA show how template-based fiber tracking can affect the estimation of variables used to quantify muscle architecture. We found, having independently varied 12 parameters that determine 3D fascicle reconstruction, that digital models of muscle architecture showed low variance in fascicle length and pennation angle. Also, when virtual models converged on the observed architecture in the dissected muscle, we found these optimized estimates of fascicle length and pennation were similar to those calculated from dissection. Regardless, caution is warranted when modifying Xfiber parameters because while the effects of certain parameters on others are additive (e.g., Minimum Trace Length, which eliminates all fibers under a specified value), others are not easily predictable (e.g., Outer Cylinder Radius). As a result, our model of muscle architecture (Fig. 3) of the starling shoulder is indeed somewhat subjective, and perhaps quantitatively suboptimal, but it does converge on aspects of the CT-apparent and dissected data suggesting this is a robust approach to visualizing and quantifying 3D muscle architecture.

Dissection-based and digital methods produced different estimates of fascicle length, pennation angle, and volume, leading to a 0.167 cm^2^ discrepancy in PCSA between methods (Table 2). However, the significance of the directions and magnitudes of these differences was not at all apparent, owing to both our sample size and to our reluctance to interpret the dissection results as ground truth. It remains to be seen whether our observed trends—that is, the Xfiber method’s underestimation of fascicle length, overestimation of volume, and roughly equivalent pennation angles relative to dissection—are generally true or are specific to our specimen. We therefore discuss the variable estimates from each method with minimal reference to each other.

#### Fascicle length

Regardless of attempted parameter combination, unrealistically long and short tracts were traced and needed pruning before quantitative analysis. The density of reconstructed tracts (adjusted via the Minimum Distance parameter) was proportional to the amount of implausible tracts in any model, and thus also to the amount of manual filtering and pruning needed to produce sufficiently accurate models. Similar results were found by Kupczik et al. (2015), who achieved greater quantitative correspondence between dissection and digital methods once their digital models were pruned to exclude tracts with lengths larger or smaller than observed in dissection, although they did not alter their digital model otherwise.

A possible bias toward selecting larger fascicles may have inflated the fascicle length estimate from dissection. We did not correct for optimal sarcomere length (Infantolino et al. 2010), which may have driven some of the difference if, for example, post-staining shrinkage also corresponded with a decrease in average sarcomere length. However, it is unknown if sarcomere length correction is meaningful in preserved and stained tissues.

#### Pennation angle

Of the three variables collected (pennation angle, fascicle length, volume), pennation angle showed the closest correspondence between methods (Table 2). In dissection, pennation angles of 30 fascicles were estimated by exposing the internal tendon and measuring the angle between tendon and fascicle. However, the sampling method, which involved dissection to expose deep aspects of the internal tendon, may not measure true pennation angle—that is, the smallest angle between tendon plane and fascicle (or myofiber) near the point of intersection. Further, the fascicle sampling and lack of 3D spatial information precluded knowledge of intramuscular pennation angle variation. Our digital method—similar to methods used in previous DiceCT fiber-tracking studies (Kupczik et al. 2015; Dickinson et al. 2018)—does produce a muscle-wide distribution of pennation angles. For muscles whose internal tendons stray from planarity, however, tract-specific values within the distribution may not correspond with actual pennation angles. For example, because the m. supracoracoideus tendon in our specimen was visibly non-planar (Fig. 6B, C), use of the *z*-axis as a proxy for the tendon plane (the “plane” from which average tract angles are measured) was not ideal. The muscle-wide distribution of pennation angles is thus influenced by the orientation of the whole muscle along the *z*-axis, with an error commensurate with the degree of planarity in the tendon. As a result, a potential advantage of our digital method over dissection may not apply to muscles with complex tendon morphology, and incorrect data distributions may underlie even plausible mean pennation angle estimates.

The most accurate technique involves measuring true fascicle-specific pennation angle—that is, the smallest angle between a tangent plane at the point of fascicle/tendon intersection and the average orientation of a fascicle for a set length from its tendinous origin. Such a procedure is used in DT-MRI (Lansdown et al. 2007) and digital microscribe (Lee et al. 2015) modeling methods and could feasibly be incorporated into CT-based fiber-tracking algorithms.

## Conclusion

Perhaps the greatest questions raised here, and in muscle architecture investigation generally, are (1) how do digital and manual methods compare to truth?, (2) are data gathered from different methods comparable?, and (3) how much discriminatory power is afforded by any method? It is unclear how much our results are specimen- or method-specific. Hence, neither the raw DiceCT image data nor the variable estimates from either method are intended for comparative use. Further sensitivity analyses and true validation against the current standard (Otake et al. 2017), whole-muscle microscribe digitization (e.g., Lee et al. 2015; Rosin and Nyakatura 2017), should be carried out, preferably on muscles with varied architectural complexity.

Overall, muscle fiber modeling using DiceCT offers a wealth of new visualization and quantitative data to other digital and non-digital methods of studying muscle architecture, especially when destructive methods are impermissible or live specimens are not available. Most significantly, CT-based algorithmic modeling methods are bound to spark renewed interest in muscle architecture and muscle–tendon morphology, especially in taxa like birds for which few muscle architecture hypotheses have been tested. While single-figure estimates of muscle architecture (e.g., mean pennation angle and PCSA) will certainly see continued use in biomechanical modeling and for purpose of comparison, the much richer, 3D representations of muscle morphology afforded by digital methods will undoubtedly prompt new and more nuanced investigations pertaining to musculoskeletal evolution, taxon-specific muscle function, and the form–function relationship of skeletal muscle across vertebrates.

## Supplementary Material

Supplementary Data
