# Supplementary material for: 3D Muscle Architecture of the Pectoral Muscles of European Starling (Sturnus vulgaris)
Source: Integr Org Biol. 2019 Feb 1;1(1):oby010. doi: 10.1093/iob/oby010 (PMC7671135; doi:10.1093/iob/oby010)

**Figure S2**. Quantitative results of the Xfiber sensitivity analysis for Curved Length (a proxy for fascicle length). The baseline run from which parameter values were increased or decreased is indicated in blue. Dots indicate the Curved Length values of each tract within a model and are summarized as standard boxplots. Dissection-derived fascicle length statistics are indicated in red along the y-axis (first quartile, median, third quartile). Parameter units are noted along the x-axis.


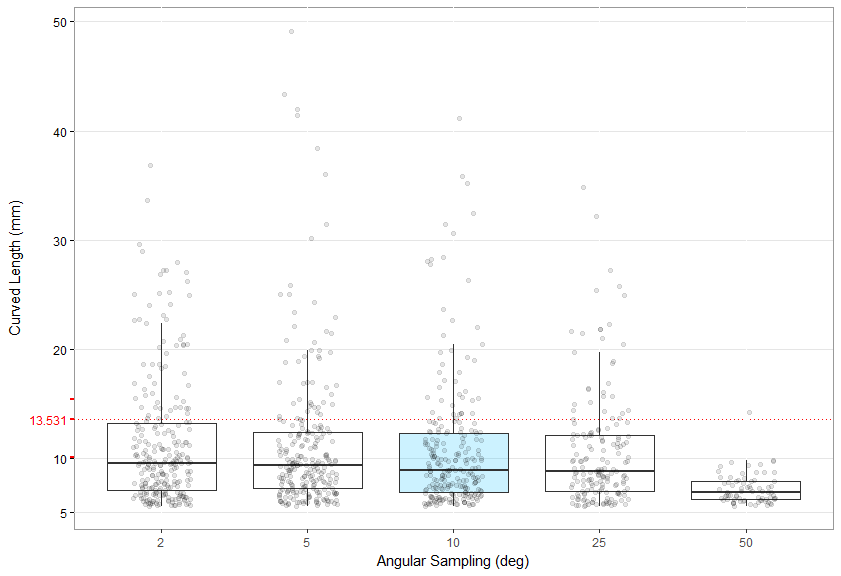


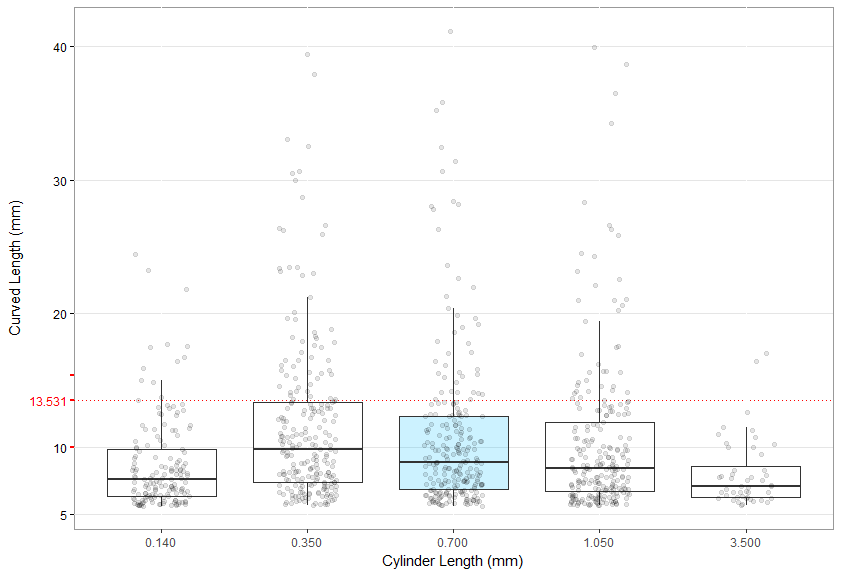


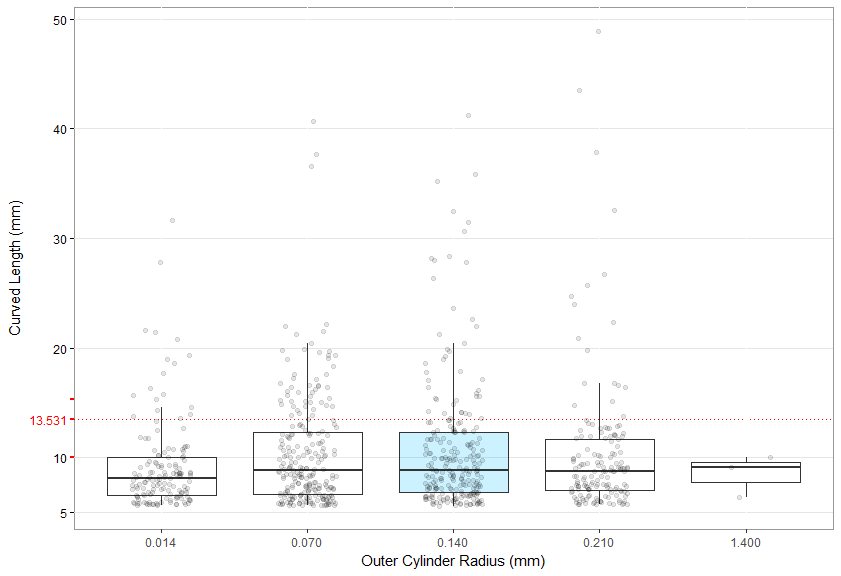


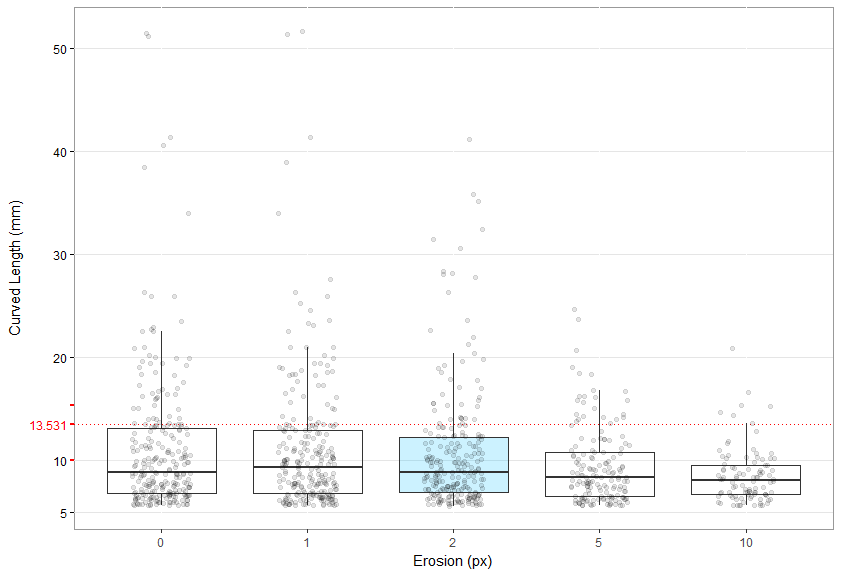


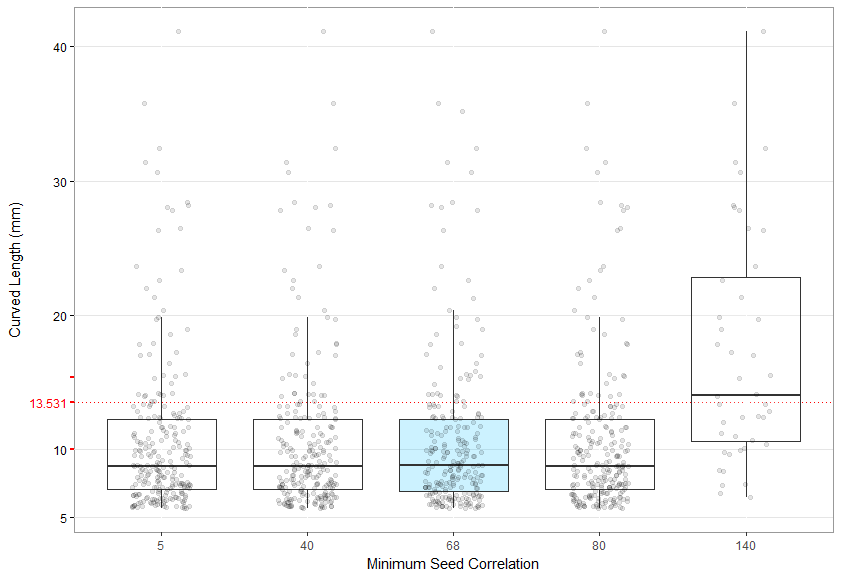


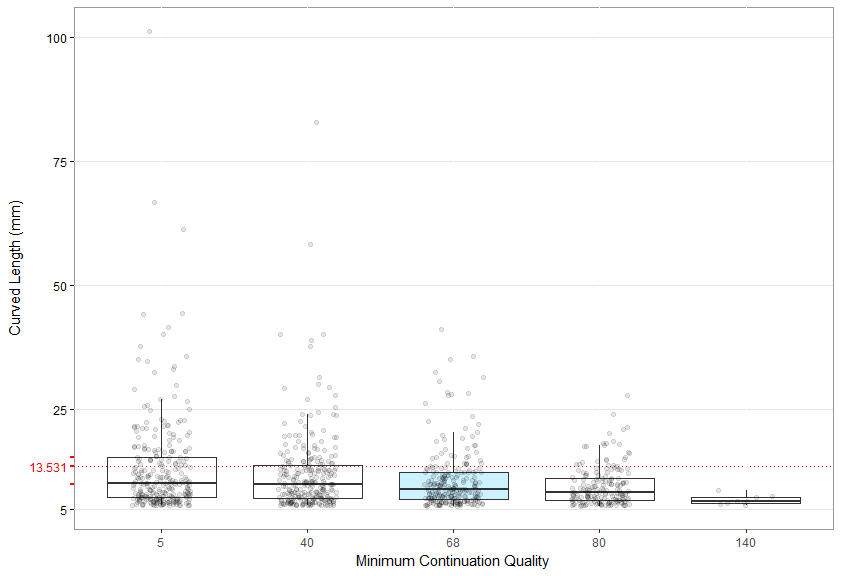


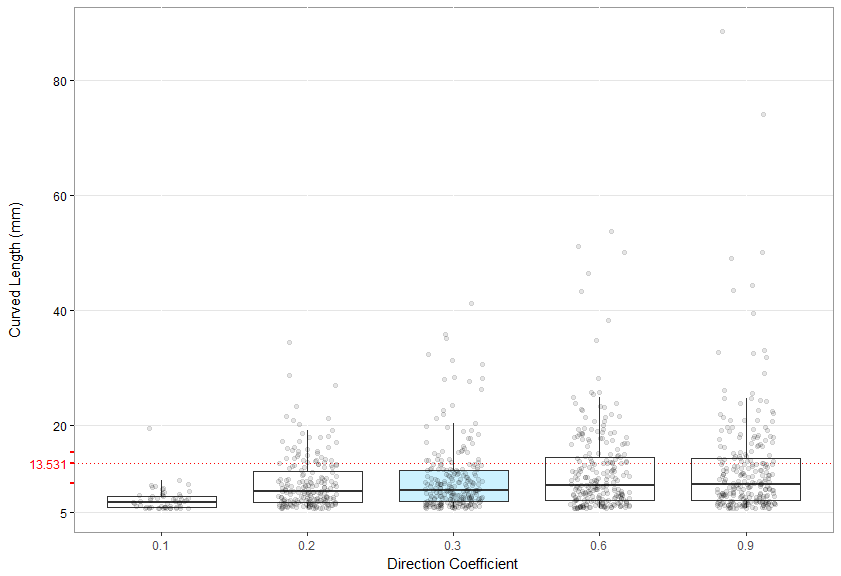


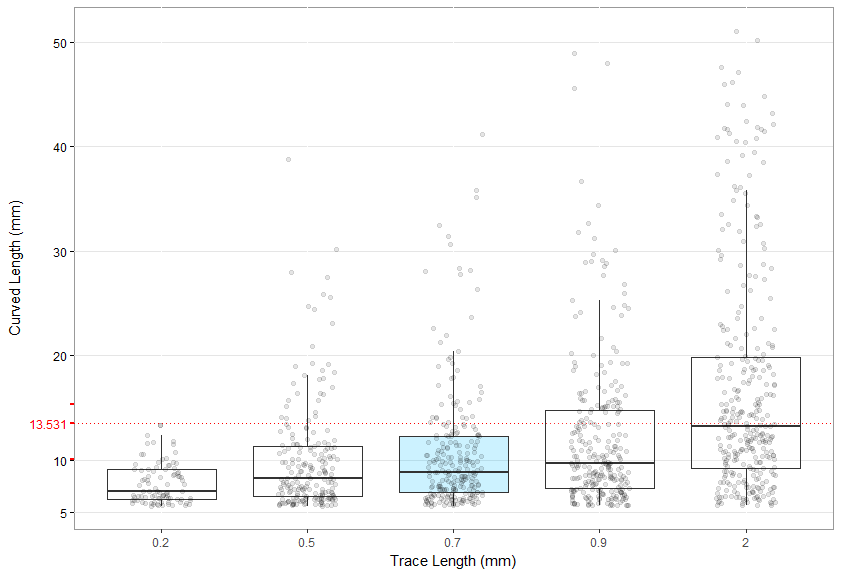


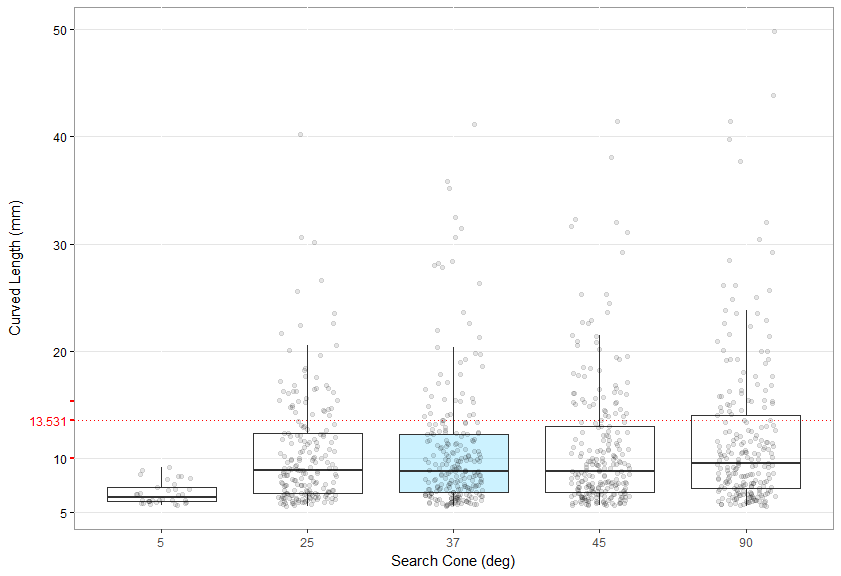


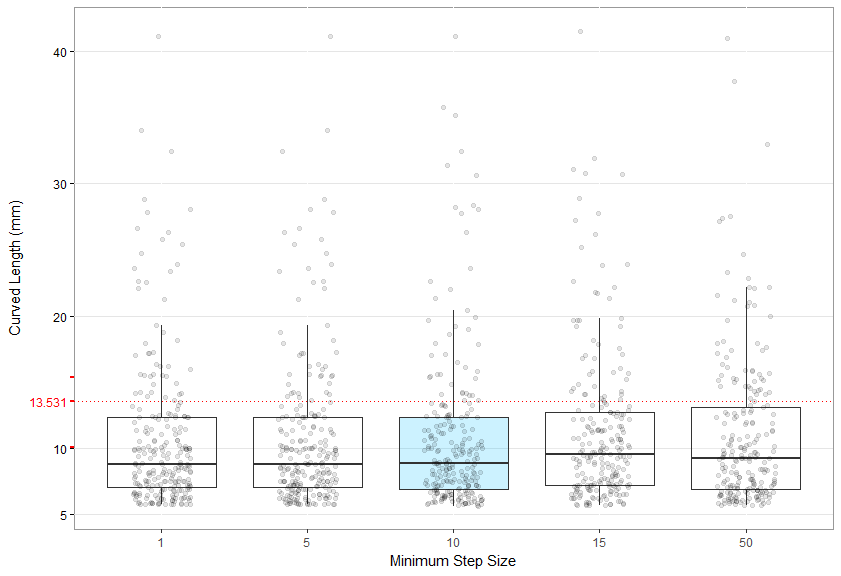


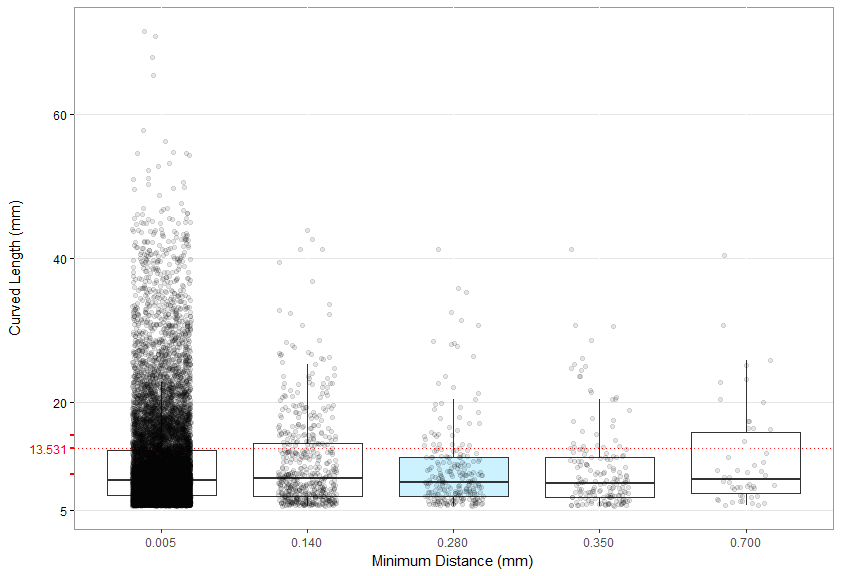


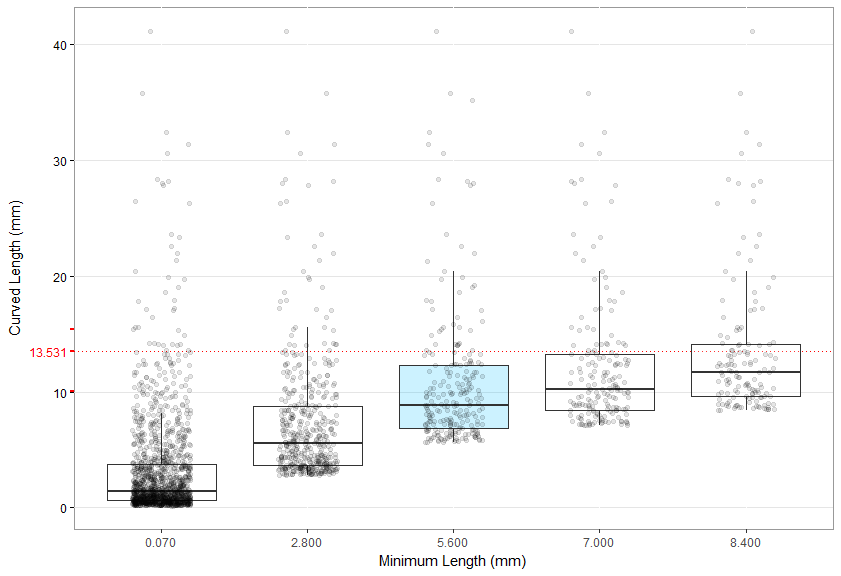

Supplement: Supplementary Data [file oby010_supp.zip › 2018_SS_IOB_FigureS2_FINAL.docx]
