# Supplementary material for: 3D Muscle Architecture of the Pectoral Muscles of European Starling (Sturnus vulgaris)
Source: Integr Org Biol. 2019 Feb 1;1(1):oby010. doi: 10.1093/iob/oby010 (PMC7671135; doi:10.1093/iob/oby010)
Supplement: Supplementary Data [file oby010_supp.zip › 2018_SS_IOB_TableS1_FINAL.docx]

Table S1. Xfiber parameter settings for complete shoulder muscle architecture model.

| Muscle | Run | Cylinder Length | Angular Sampling | Cylinder Mask Radius | Cylinder Outer Radius | Erosion | Minimum Seed Correlation | Minimum Continuation Quality | Direction Coefficient | Search Cone Length | Search Cone Angle | Search Cone Minimum Step Size | Minimum Distance | Minimum Length |
| --- | --- | --- | --- | --- | --- | --- | --- | --- | --- | --- | --- | --- | --- | --- |
| PECT | 9 | 1400 | 10 | 150 | 140 | 2 | 68 | 45 | 0.3 | 1400 | 37 | 10 | 280 | 5600 |
| SC | 10 | 700 | 2 | 150 | 140 | 0 | 68 | 45 | 0.3 | 700 | 37 | 10 | 280 | 5600 |
| CBCA | 5 | 1000 | 10 | 90 | 85 | 1 | 68 | 45 | 0.3 | 1000 | 37 | 10 | 200 | 5600 |
| STC | 2 | 700 | 10 | 85 | 80 | 1 | 68 | 40 | 0.22 | 700 | 37 | 10 | 150 | 5600 |
| SBCD | 5 | 800 | 10 | 90 | 85 | 1 | 68 | 45 | 0.3 | 700 | 37 | 10 | 280 | 5600 |
| SHCA | 3 | 1000 | 10 | 115 | 110 | 1 | 68 | 45 | 0.3 | 1000 | 37 | 10 | 280 | 5000 |
| HT | 1 | 900 | 10 | 95 | 90 | 1 | 68 | 45 | 0.3 | 700 | 37 | 10 | 280 | 5600 |
| BB | 3 | 350 | 10 | 75 | 70 | 0 | 68 | 48 | 0.22 | 350 | 37 | 10 | 50 | 1000 |
| SHCR | 1 | 700 | 10 | 75 | 70 | 0 | 68 | 45 | 0.3 | 700 | 37 | 10 | 200 | 5600 |
| ST | 1 | 900 | 10 | 95 | 90 | 1 | 68 | 45 | 0.3 | 900 | 37 | 10 | 280 | 5600 |
| DM | 2 | 1000 | 5 | 110 | 100 | 1 | 68 | 45 | 0.3 | 1000 | 37 | 10 | 280 | 5600 |
| DMI | 1 | 700 | 10 | 75 | 70 | 0 | 68 | 45 | 0.3 | 700 | 37 | 10 | 200 | 5600 |
| SBS | 1 | 700 | 10 | 80 | 75 | 1 | 68 | 45 | 0.3 | 700 | 37 | 10 | 200 | 3000 |
| PP | 1 | 700 | 10 | 75 | 65 | 1 | 68 | 45 | 0.3 | 700 | 37 | 10 | 200 | 5600 |
